# Effect of porosities of bilayered porous scaffolds on spontaneous osteochondral repair in cartilage tissue engineering

**DOI:** 10.1093/rb/rbv001

**Published:** 2015-03-06

**Authors:** Zhen Pan, Pingguo Duan, Xiangnan Liu, Huiren Wang, Lu Cao, Yao He, Jian Dong, Jiandong Ding

**Affiliations:** ^1^State Key Laboratory of Molecular Engineering of Polymers, Department of Macromolecular Science, Advanced Materials Laboratory, Fudan University, Shanghai 200433, China; ^2^Department of Orthopaedic Surgery, The First Affiliated Hospital of Nanchang University, Nanchang, 330006, China; ^3^Department of Orthopaedic Surgery, Zhongshan Hospital, State Key Laboratory of Molecular Engineering of Polymers, Fudan University, Shanghai 200032, China

**Keywords:** bilayered scaffold, porosity, mesenchymal stem cell, osteochondral defect, PLGA, cartilage tissue engineering

## Abstract

Poly(lactide-*co*-glycolide)-bilayered scaffolds with the same porosity or different ones on the two layers were fabricated, and the porosity effect on *in vivo* repairing of the osteochondral defect was examined in a comparative way for the first time. The constructs of scaffolds and bone marrow-derived mesenchymal stem cells were implanted into pre-created osteochondral defects in the femoral condyle of New Zealand white rabbits. After 12 weeks, all experimental groups exhibited good cartilage repairing according to macroscopic appearance, cross-section view, haematoxylin and eosin staining, toluidine blue staining, immunohistochemical staining and real-time polymerase chain reaction of characteristic genes. The group of 92% porosity in the cartilage layer and 77% porosity in the bone layer resulted in the best efficacy, which was understood by more biomechanical mimicking of the natural cartilage and subchondral bone. This study illustrates unambiguously that cartilage tissue engineering allows for a wide range of scaffold porosity, yet some porosity group is optimal. It is also revealed that the biomechanical matching with the natural composite tissue should be taken into consideration in the design of practical biomaterials, which is especially important for porosities of a multi-compartment scaffold concerning connected tissues.

## Introduction

Cartilage repair is a challenging topic in regenerative medicine [1–7]. Articular cartilage is hard to regenerate due to its avascularity, less migration of chondrocytes surrounded by their extracellular matrix and the limited proliferation of mature chondrocytes, etc. [1]. Abrasion arthroplasty, microfracture, autologous chondrocyte implantation and osteochondral autologous transfer have been applied to repair cartilage defects; yet there is still no completely successful and universally accepted approach for the treatment of damaged articular cartilage over a critical defect size, especially for large osteochondral injuries [8]. An osteochondral defect involves damage to both the full-thickness articular cartilage and a part of the underlying subchondral bone, leading to an osteoarthritic degenerative change due to the mechanical instability of the joint [9]. Because the mere replacement of joint cartilage is frequently faced with difficulty in tightly connecting with its subchondral bone, the osteochondral repair is very meaningful for cartilage tissue engineering [9–14].

**Figure 1.**
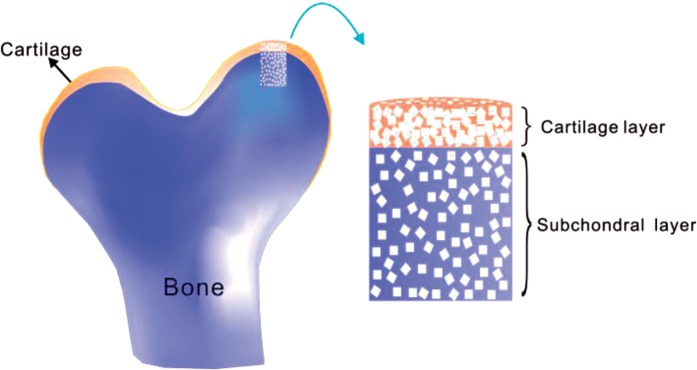
Schematic presentation to use a bilayered scaffold to restore the osteochondral defect in the knee joint.

The articular cartilage and underlying subchondral bone have different intrinsic structures and physiological functions, so that single-layer tissue engineering scaffolds with homogenous properties may not be ideal to support the metabolic and morphogenic activities of multiple cell types. Bilayered scaffolds with discrete regions or compartments have thus been designed to mimic appropriate biochemical, physical and mechanical conditions of cartilage and bone [1, 15–20]. A bilayered scaffold is schematically presented in Fig. 1. The applications of bilayered scaffolds constitute a hot aspect in the fields of biomaterials, tissue engineering and regenerative medicine.

Among basic scaffold parameters, porosity plays a critical role in tissue engineering [21, 22]. Although many reports about cartilage repair pertinent to bilayered scaffolds have emerged recently, the porosity effect has not yet been experimentally examined. In many cases, the same porosity was made for both cartilage and bone layers [23–25]; in all cases of different porosities, just one combination group was examined in one publication [3, 12, 26, 27]. We cannot find any report concerning even two groups of porosities for bilayered scaffolds in one publication of cartilage tissue engineering, to the best of our knowledge. The porosity effect has been examined in cartilage tissue engineering using single-layer scaffolds [28] and other tissue engineering cases such as osteogenesis [29]. Nevertheless, the basic data about the effect of scaffold porosity on *in vivo* tissue repairing are still rather limited. In fact, some excellent discussion about porosity effects on tissue engineering was based mainly upon experimental results of different porosities from different experiments of different research groups [21]. Hence, it might be helpful to design a series of comparable experiments to focus upon the *in vivo* porosity effect while keeping the other scaffold parameters fixed, which triggered our present investigation.

In this study, poly(lactide-*co*-glycolide) (PLGA) was used as the scaffold matrix. It is a common and important biodegradable polyester [10, 30–33]. Foam-like PLGA scaffolds for either cartilage layer or bone layer were fabricated using salt particles as porogen and using room-temperature compression moulding for shaping; and the approach for single-layer scaffolds has been reported by us [34]. The pore size and porosity are ready to be controlled by the porogen size and content [22, 35, 36]. To enable this study, we developed a facile approach to stick the two PLGA scaffolds by dichloromethane, and the bilayered scaffolds were designed of a given reasonable pore size (200–300 μm) yet three combination groups of porosities, as indicated in Table 1. Considering that a very high porosity must lead to a mechanically weak scaffold and a low porosity can even not guarantee the interconnectivity of pores for common foam-like PLGA scaffolds, we examined only the reasonable range of moderate porosity, to reduce the number of sacrificed animals.

**Table 1.** Porosity combination of the bilayered PLGA scaffolds.

Bilayered PLGA scaffold	Porosity (cartilage layer)	Porosity (subchondral layer)
Scaffold A	92%	77%
Scaffold B	85%	85%
Scaffold C	77%	92%

A New Zealand white rabbit model was used in our animal experiment, and bone marrow-derived mesenchymal stem cells (BMSCs) served as seeding cells. An osteochondral defect (4 mm in diameter and 5 mm in depth) was created using a surgical drill on the femoral condyle of rabbits. Bilayered scaffolds with and without cells were then transplanted into the osteochondral defects of rabbits, and the repairing efficacy was checked after 6 and 12 weeks.

## Materials and methods

### Preparation of bilayered PLGA scaffolds

Porous scaffolds were fabricated by ‘room-temperature’ compression moulding/particulate leaching method [34, 37, 38]. PLGA (Purac Co., The Netherlands) with a copolymer ratio of 85/15 (lactide/glycolide)was dissolved in dichloromethane and then mixed with salt particles (200–300 µm) to form a paste-like mixture. The mixture was pressed into a mould and kept under pressure for 24 h. After the mould was released, the shaped cylinder mixture was obtained.

Cylinder mixtures with different porogen contents were stuck by dichloromethane under pressure and cut into 4 mm in diameter and 5 mm in thickness (cartilage layer: 1 mm, subchondral layer: 4 mm). After leaching porogen by deionized water, we obtained the bilayered PLGA scaffolds.

The scaffold porosity was well controlled by the content of porogen. We first prepared a series of single-layer scaffolds with varied weight faction of porogen. The porosities of those single-layer scaffolds were measured by a liquid replacement method [34]. Then a relation between porogen content and the resultant porosity was set up. For bilayered scaffolds, the porosities in each layer in the bilayered scaffolds were calculated by the porogen content and the pre-set calibration.

### Mechanical testing

The compressive moduli of the scaffolds were characterized by measurement of stress–strain curves at the room temperature, similar to our previous work [39]. The samples were tested on an SANS CMT4104 testing machine. Cylindrical scaffolds of 4 mm in diameter and 5 mm in height were compressed at 6.0 mm/min till 60% strain or facture.

### Isolation and culture of BMSCs

Allogenic BMSCs were isolated from 8-week-old New Zealand white rabbits. Bone marrow (5 ml) was aspirated from the iliac crests of rabbits and subsequently cultured according to our previous protocol [40]. Briefly, the isolated cell suspension was seeded in T-25 ﬂasks (Corning, USA) and cultured in 5 ml complete medium. The culture medium consisted of Dulbecco’s modified Eagle’s medium with low glucose (DMEM-LG, Gibco, USA) supplemented with 10% fetal bovine serum (Gibco, Australia), 100 mg/ml streptomycin and 100 U/ml penicillin. Cell clusters grew into colony-forming fibroblast-like cells, with medium replaced every 3 days. The cells were subcultured to the third passage.

### Cell labelling

BMSCs were tracked by 1,1-dioctadecyl-3,3,3*'*,3*'*-tetramethylindocarbocyanine perchlorate (DiI, Invitrogen, USA). This membrane-bound fluorescent dye exhibits very low cell toxicity and does not compromise cell viability and differentiation potential. Cells of passage 3 were stained by DiI according to the manufacturer’s protocol. DiI-labelled cells were resuspended in the complete medium for further use. The sample was observed on an inverted ﬂuorescence microscope (Olympus IX51), and the labelling efficiency was checked by flow cytometry (BD FACSAria II).

### Seeding of BMSCs into scaffolds

The scaffolds with ethylene oxide sterilization were placed in each well of 24-well culture plates (Corning, USA) and incubated in DMEM-LG overnight for seeding effectively. The third-passage BMSCs were used, and aliquots of 20 µl cell suspensions (5 × 107 cells/ml) were injected into the scaffolds using a 1 ml syringe. The cell-seeded scaffolds (1 × 106 cells/scaffold) were incubated in standard conditions for 2 h to make cells adhere well to the scaffold and incubated for 1 week *in vitro*. The cell-free scaffolds were incubated under the same conditions.

Scanning electron microscopy (SEM) (TS5136MM, TESCAN) was used to observe the interior surfaces of scaffolds with cells. After culture for 1 week, the cell-seeded scaffolds were fixed in 2.5% glutaraldehyde at 4°C for 24 h. Then, the samples were dehydrated in graded ethanol, and eventually a critical-point drying was made. Gold was sputtered to coat the surfaces prior to SEM observations.

### Surgical implantation

All treatments of animals were performed in strict accordance with the guidelines of and approved by the Institutional Animal Care and Use Committee of Fudan University. Twenty-four skeletal mature New Zealand white rabbits (5–6 months old) of 2.9–3.2 kg were used in the study. After 1-week acclimation, the rabbits were anaesthetized with ketamine hydrochloride (35 mg/kg). Amedial parapatellar incision was made on the bilateral knee joints after being disinfected. The dissection continued until the femoral condyle was exposed. An osteochondral defect (4 mm in diameter and 5 mm in depth) centred on the femoral condyle was created using a surgical drill bit (customized) with scale marks. Thereafter, the cell-seeded scaffolds and the corresponding cell-free scaffolds were implanted into the medial and lateral condyles in each joint by press-fitting.

Rabbit knees were assigned randomly to the implants of three pairs (the cell-seeded scaffold and the corresponding cell-free scaffold for one pair) (*n* = 6 for each scaffold group). Additionally, for the other knee in the same rabbit, the autologous osteochondral plug was re-implanted into the medial condyle in the group of ‘normal’, and the defect of lateral condyle was kept empty as the group of ‘blank’.

After implantation, the surgical incision was closed layer by layer. The rabbits were fed tap water and food, kept in separate cages and allowed to move freely. Gentamycin (4 mg/kg) was injected intramuscularly once a day during the initial postsurgery 3 days.

### Tissue retrieval and histological analysis

The rabbits were sacrificed by injection of excess ketamine hydrochloride at 6 and 12 weeks postoperatively (12 rabbits for each time point). After photographing the joints, some 6-week samples were divided into two halves. One-half samples were fixed in 4% formalin, decalcified, then embedded in paraffin and sectioned at 4 μm using a rotary microtome (Leica RM2235, Germany). The sections were stained with haematoxylin and eosin (H&E), toluidine blue and safranin O/fast green. The paraffin sections were further used for immunohistochemical examinations. The sections were blocked with 10% goat serum and then incubated with 10 μg/ml anti-collagen type I or collagen type II mouse monoclonal antibody (EMD Millipore, USA) at 4°C overnight. After washing with phosphate-buffered saline, the sections were incubated with biotinylated goat anti-mouse secondary antibody (DAKO, Carpinteria, CA) at 37°C for 20min. Finally, the samples were incubated in 3,3*′*-diaminobenzi-dine tetrahydrochloride (DAB) solution (0.5 mg/ml, with 0.01% H_2_O_2_ and Tris-HCl buffer solution as solvent, pH 7.6).

The other half was for fluorescence observations. The samples were quickly frozen by liquid nitrogen and then embedded in optimum cutting temperature compound (10.24%w/w poly(vinyl alcohol), 4.26%w/w poly(ethylene glycol)and 85.50%w/w nonreactive ingredients, Tissue-Tek, CA). The specimens were cryosectioned at 10 -µm intervals under a freezing microtome (Leica CM1850, Germany); the cryosections were stained by 4*′*,6-diamidino-2-phenylindole (DAPI, Sigma) and then observed on an upright ﬂuorescence microscope (Olympus BX51). DiI-labelled BMSCs were examined on the microscope as well.

At 12 weeks, the samples were also divided into two halves. One half was used to histological staining and immunohistochemistry staining as described in the former paragraph. The histologic grading scales as described by Wakitani *et al.* [41] were used to evaluate the quality of the repaired tissue by two skilful individuals in a double-blind way. The other half samples were quickly frozen by liquid nitrogen for real-time polymerase chain reaction (PCR) assay.

### Real-time PCR assay

As mentioned above, some specimens of 12 weeks after implantation were harvested for real-time PCR. Total RNA was isolated using Trizol reagent (Invitrogen, USA) according to the manufacturer’s protocol. The cDNA was reverse-transcribed with a Reverse Transcription System (TaKaRa). The primer sequences specific for the target genes for real-time PCR are listed in Table 2, along with the internal control of the gene glyceraldehyde-3-phosphate dehydrogenase (GAPDH). The real-time PCR measurements of collagen type I, collagen type II, aggrecan and GAPDH were carried out as previously described [42], which was performed in an ABI 9700 real-time PCR system using Brilliant SYBR Green QPCR master mix (TaKaRa) at 90°C for 15 s and at 60°C for 60 s. The ﬂuorescence intensity was recorded for 40 cycles.

**Table 2.** Nucleotide primers used for real-time PCR.

Genes	Forward primer sequence (5′-3′)	Reverse primer sequence (5′-3′)	Size (bp)
Collagen type II	GAGCAGCAAGAGCCAGAAGCA	GGAGCCCTCAGTGGACAGCA	148
Collagen type I	GCAGGGCTCCAATGATGTT	AAGGAAGGGCAAACGAGAT	151
Aggrecan	CAGAACTTTGGTAGAATCCGTAA	CCAGAATGGGCTCCAGACAC	123
GAPDH	AGCACCAGAGGAGGACGAG	GGATGGAAACTGTGAAGAGGG	100

### Statistical analysis

Data were expressed as mean ± standard deviation. Statistical analysis was performed using Student’s *t*-test or one-way analysis of variance (ANOVA). A value of *P* < 0.05 was considered statistically significant.

## Results

### Fabrication of bilayered PLGA scaffolds

**Figure 2.**
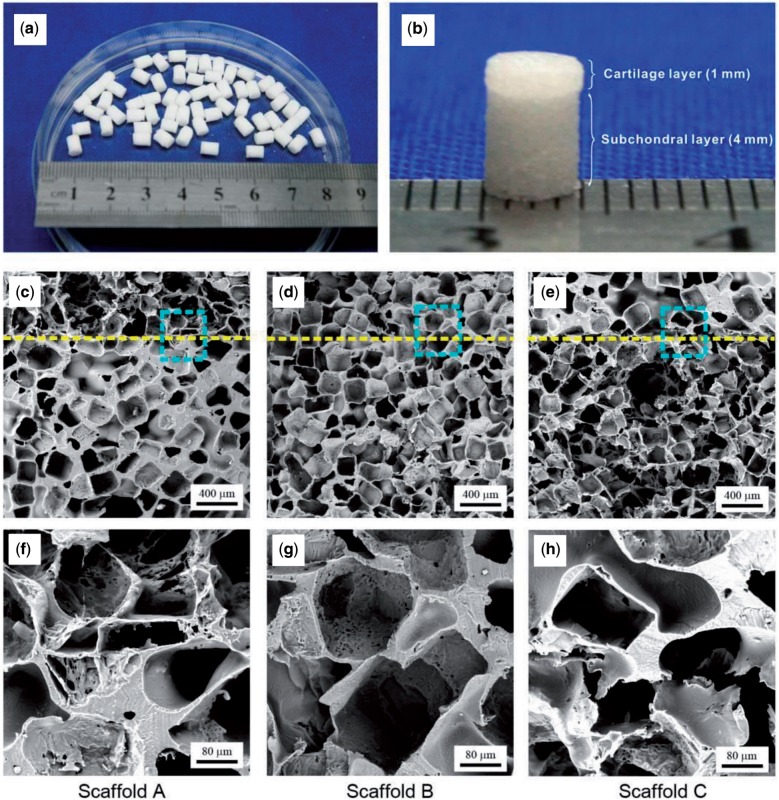
Images of prepared bilayered PLGA scaffolds. (**a**, **b**) Gross view of scaffold A. (**c–h**) SEM micrographs of three groups of bilayered scaffolds. The dashed lines indicate the border of the two layers. The lower row displays the corresponding magnified images of the squared regions in the middle row.

The bilayered scaffolds with 4 mm of diameter and 5 mm of the total thickness were fabricated by us, with some resultant scaffolds shown in Fig. 2a. Three groups of bilayered scaffolds were designed in our experiments, as listed in Table 1. A typical bilayered scaffold (scaffold A) is shown in Fig. 2b. The SEM micrographs of all three scaffolds are presented in Fig. 2c–h, and microscopic pore structures in the bilayered scaffolds are well confirmed.

### Mechanical properties of bilayered scaffolds

**Figure 3.**
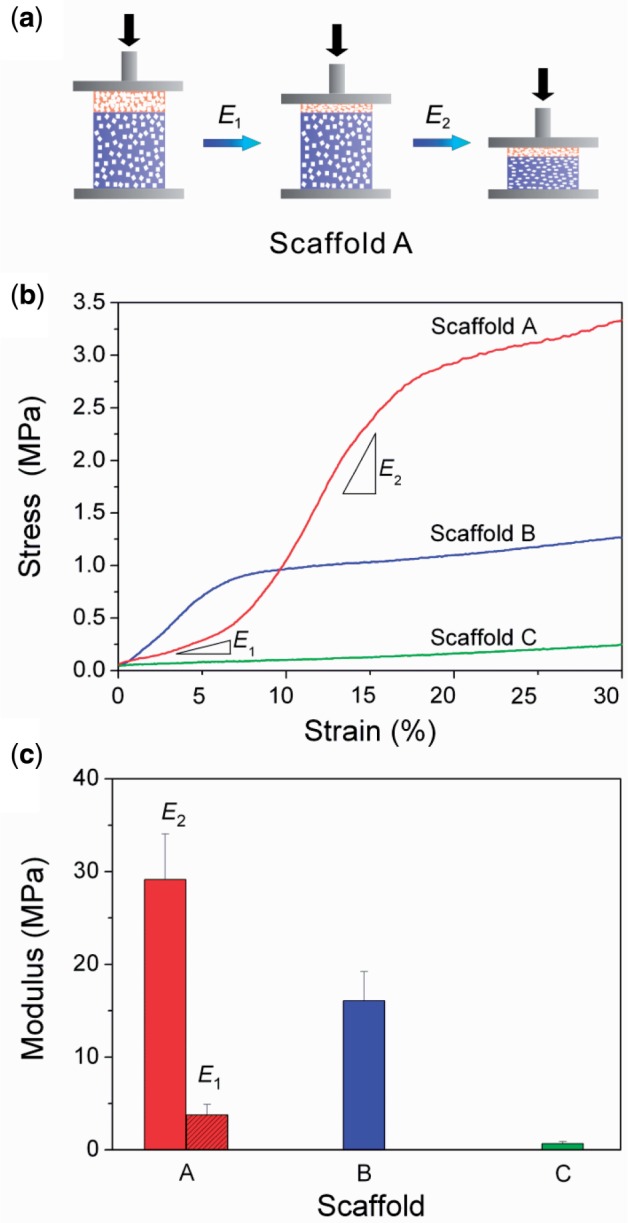
(**a**) Schematic presentation of the compressing process of Scaffold A. *E*1 represents the compressive modulus of the cartilage layer and *E*2 for the subchondral layer. (**b**) Typical stress–strain curves of three scaffolds. (**c**) The compressive moduli of three groups of bilayered scaffolds. *n* = 6 for each group.

The mechanical properties of our bilayered scaffolds were measured, as schematically presented in Fig. 3a. The slope in the linear elastic region gave the compressive modulus *E*. For scaffold A, two slopes were observed in the stress–strain curves: *E*1 (3.8 ± 1.1 MPa) reflects the modulus of the cartilage layer with the initial porosity 92% and *E*2 (29.1 ± 5.0 MPa), of the subchondral layer with the initial porosity 77%. Scaffold B has the same porosity in both layers and thus exhibited only one elastic region with the compressive modulus 16.1 ± 3.2 MPa. The second turning point in the stress–strain curve of scaffold A and the first turning point for scaffold B in Fig. 3b indicate the flexure point, which is common in highly porous PLGA scaffolds [39, 43]. The flexure was not observed in the cases of scaffold C in Fig. 3b. For scaffold C, the major part of the bilayered scaffold was of the high porosity (4 mm, porosity: 92%); only one slope was observed in Fig. 3b till the maximum strain (60%) we examined, resulting in compressive modulus 0.7 ± 0.2 MPa as shown in Fig. 3c.

### Implantation of bilayered scaffolds to regenerate osteochondral defects

**Figure 4.**
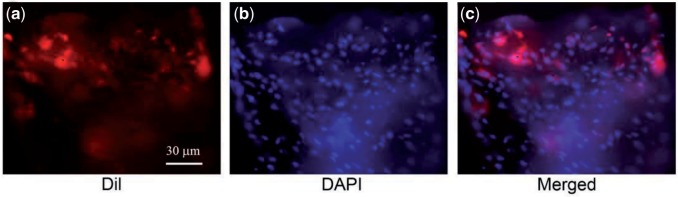
Fluorescence micrographs of neo-tissues 6 weeks after implantation of porous scaffolds seeded by BMSCs with their cell membranes labelled by DiI. (**a**) DiI-labelled cells (red) in the neo-tissue zone; (**b**) the same viewing field with cell nuclei stained by DAPI (blue); (**c**) the merged image of (a) and (b).

We confirmed the good adhesion of BMSCs on the pore walls of the PLGA scaffolds *in vitro* (data not shown). To probe the migration of external cells *in vivo*, we labelled BMSCs by DiI. The labelling efficiency was about 97% as determined by flow cytometry. These cells were seeded into the bilayered scaffolds and cultured *in vitro* for 1 week. Then, the cell-scaffold constructs were implanted into the osteochondral defects. Six weeks after implantation, fluorescence of BMSCs with their cell membranes labelled by DiI in porous scaffolds was still detectable, as shown in Fig. 4.

**Figure 5.**
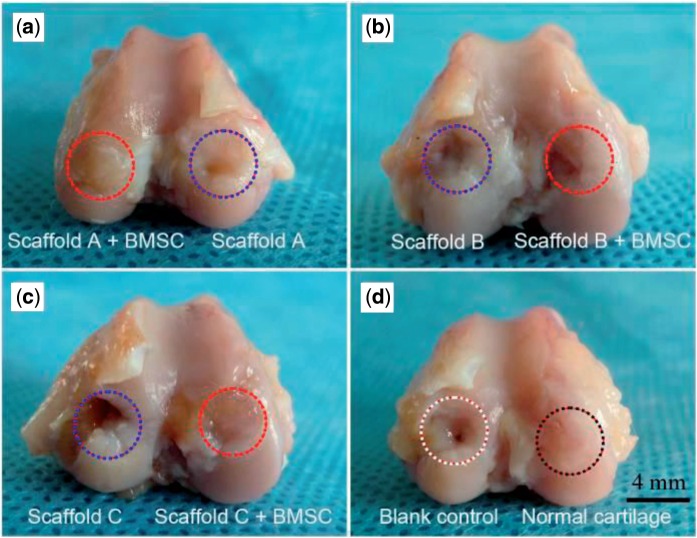
Gross views of regenerative tissues 12 weeks after implantation. Both medial and lateral condyles of knee joints of New Zealand white rabbits were drilled and then repaired by implanting PLGA scaffolds with or without external BMSCs. The blank control refers to neither scaffold nor external cells; the group of ‘normal’ means re-implantation of autologous osteochondral plug.

**Figure 6.**
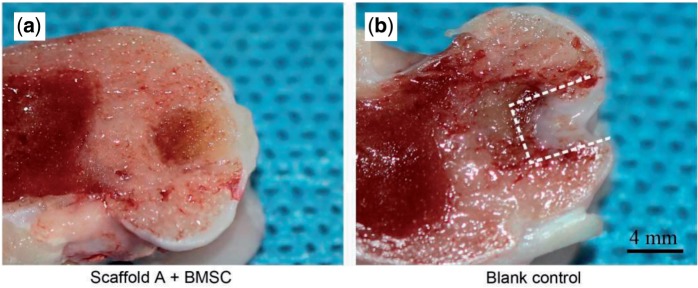
Cross-section views of the regenerative osteochondral tissue repaired by scaffolds A with external BMSCs (**a**) and the blank control (**b**). The dashed lines indicate the border between the neo-tissue and the native cartilage.

The critical defect size of joint cartilage for rabbit is 3 mm [44], and thus the defect in this study (4 mm in diameter and 5 mm in depth) is over critical. The blank control was thus not healed, as confirmed in Figs. 5d and 6b. The best gross appearance was observed in the group of scaffold A with BMSCs seeded, as seen in Fig. 5a. Even the border between the neo-cartilage and the native cartilage became obscure after12-week implantation, as seen from the cross-section view in Fig. 6a.

### Histological examinations and scoring

**Figure 7.**
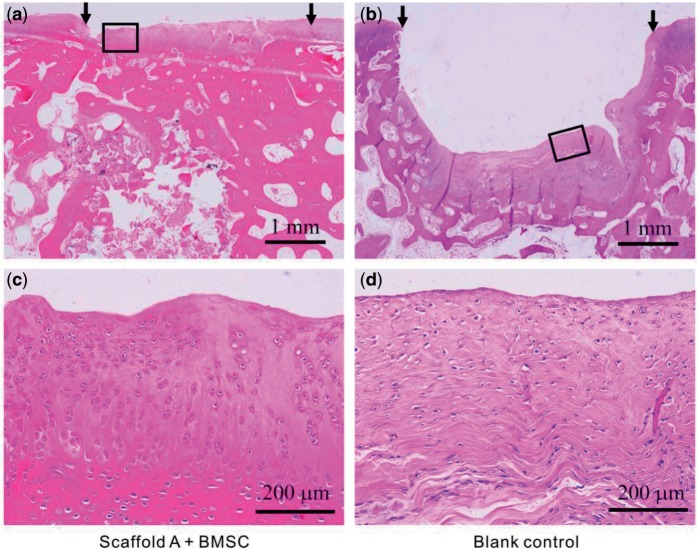
Histological images of reparative tissues 12 weeks after implantation (H&E staining).(**a**) Tissues repaired by scaffold A with BMSCs; (**b**) blank control with no implantation; (**c**) and (**d**) are the magnified images of the rectangles in (a) and (b), respectively. The arrows indicate the initial defect site.

Some histological examinations were carried out, including H&E staining, toluidine blue and immunohistochemical staining. After implantation for 12 weeks, defects repaired by scaffold A with cells were fully filled with the regenerative tissue (Fig. 7a). The neo-cartilage, whose thickness was quite close to the normal cartilage, integrated well with the adjacent host tissue, led to an indistinct interface. Seen from Fig. 7c, cells in the upper layer were predominantly round and lacunated, which are characteristic for a hyaline cartilage. In contrast, the defects without scaffolds left a big gap in the site. Cells in the blank control were predominantly fibroblast-like spindle cells without lacunae and oriented parallel to the surface of the subchondral bone (Fig. 7b and d).

**Figure 8.**
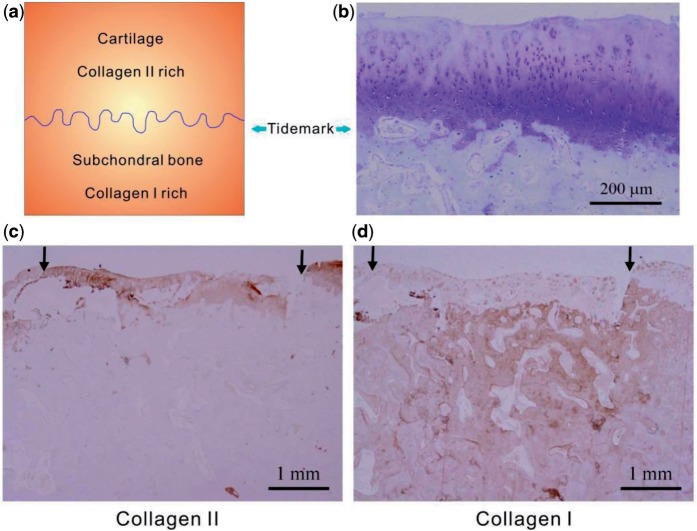
(**a**) Schematic presentation of the tidemark between cartilage and subchondral bone and the rich collagen types in the two regions. (**b–d**) Images of cross sections of reparative tissues 12 weeks after implantation of scaffold A and external BMSCs, histologically stained by toluidine blue for cartilage (b), immunohistochemically stained for collagen type II (c) and collagen type I (d). The arrows indicate the initial defect site.

The regenerative cartilage by scaffold A with cells at 12 weeks postoperatively showed a constituted tidemark approximately aligned with the native osteochondral junction, and the tidemark was visualized well after toluidine blue staining (Fig. 8b). Similar to the native cartilage and the subchondral bone, tissues repaired by cell-seeded scaffolds A showed abundant collagen type II in the upper layer and rich collagen type I in the subchondral layer visualized after the immunohistochemical staining (Fig. 8c and d).

We then employed the Wakitani’s standard [41] to score the regenerative efficacy. Five categories were taken into consideration. They were cell morphology (such as mostly hyaline cartilage, mostly fibrocartilage or mostly non-cartilage), matrix-staining or metachromasia (such as normal compared with host adjacent cartilage or markedly reduced), surface regularity (such as smooth >3/4 or irregular 1/4–1/2), thickness of cartilage (such as >2/3or <1/3) and integration of donor with host adjacent cartilage (such as both edges integrated or one edge integrated). The scoring standard is listed in Table 3. The theoretical score lies between 0 and 14. The double-blind evaluation by two independent and skilled researchers resulted in non-perfect and objective scores even for the control groups of ‘normal’ and ‘blank’.

**Table 3.** Histological grading criteria for the regenerative cartilage.

Category	Points
Cell morphology	
Hylaline cartilage	0
Mostly hylaline cartilage	1
Mostly fibrocartilage	2
Mostly non-cartilage	3
Non-cartilage only	4
Matrix-staining (metachromasia)	
Normal (compared with host adjacent cartilage)	0
Slightly reduced	1
Markedly reduced	2
No metachromatic stain	3
Surface regularity	
Smooth (>3/4)	0
Moderate (>1/2–3/4)	1
Irregular (1/4–1/2)	2
Severely irregular (<1/4)	3
Thickness of cartilage	
>2/3	0
1/3–2/3	1
<1/3	2
Integration of donor with host adjacent cartilage	
Both edges integrated	0
One edges integrated	1
Neither edge integrated	2
Total maximum	14

**Figure 9.**
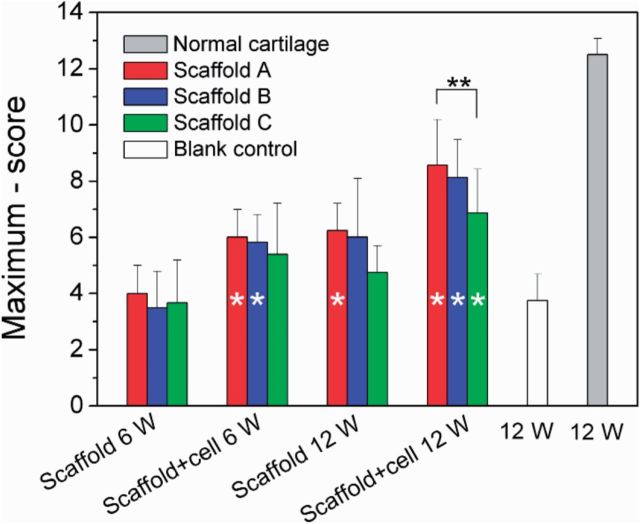
Histological scores according to Wakitani’s standard for reparative tissues of indicated groups. The vertical coordinate is the score subtracted by the maximum ‘14’. For each group, *n* = 6. Although the group of ‘normal’ refers to 12 weeks after re-implantation of the autologous osteochondral plug, the group of ‘blank’ describes 12–25 weeks after generating the osteochondral defect (4 mm in diameter and 5 mm in depth) in rabbit knees without any treatment by scaffolds or BMSCs. The hollow single asterisks in some columns indicate a significant difference compared with the group of blank with *P* < 0.05. We also made comparison between three scaffolds under a given condition (the same clusters in the histogram), and a significant difference between any two scaffolds with *P* < 0.05 is marked by ‘**’ above the corresponding columns.

The global scores of all groups are shown in Fig. 9. According to the original Wakitani’s standard, the least score means the best regenerative efficacy. Here, we suggest the maximum minus score as the normal coordinate, and thus a higher column in Fig. 9 indicates a better efficacy. At 12 weeks, all scaffolds with cells exhibited better effects compared with the blank group; the groups of scaffolds A and B with seeding cells at 6 weeks also exhibited more significant cartilage regeneration than the blank group at 12 weeks. So, PLGA porous scaffolds fabricated in this work have a definitely positive efficacy to help osteochondral healing, and the addition of external BMSCs is helpful for cartilage repairing.

The mean values of scores of scaffold A are always optimal among every comparable group clusters in Fig. 9. Although all the three scaffolds are acceptable for osteochondral repairing, the group of scaffold A with BMSCs resulted in the best cell morphology, matrix-staining, surface regularity, thickness of cartilage and integration of donor with host adjacent cartilage.

### Analysis of gene expression

**Figure 10.**
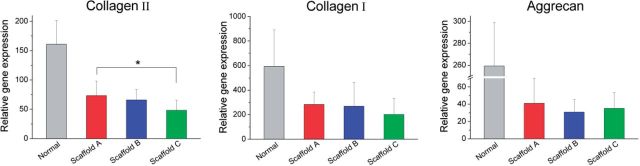
Gene expression levels of reparative tissues relative to GAPDH assessed with real-time PCR. All the scaffolds examined here were seeded with BMSCs, and the measurements were done 12 weeks after implantation. For each group, *n* = 6. *Significant difference in comparison between any two of the three scaffold groups with *P* < 0.05.

The genes pertinent to cartilage and bone were further detected by real-time PCR. According to Fig. 10, the expression level of collagen II in the group of scaffold A with cells is significant higher than that of the cell-seeded scaffold C after 12 weeks of implantation. Because of large data scattering, collagen I did not exhibit such a significant difference. Nevertheless, all three genes (collagen I, collagen II and aggrecan) resulted in the highest mean values in the group of scaffold A with cells.

## Discussion

### Successful restoring of osteochondral defects by bilayered PLGA scaffolds

The regeneration of osteochondral defects is scientifically challenging and clinically demanding, due to the insufficient natural healing of the defects and the unsuccessful clinical treatments [1, 8]. Tissue engineering and regenerative medicine provides an alternative method to restore the osteochondral defects [9, 24, 30]. In recent years, bilayered scaffolds have been tried to mimic different intrinsic structures and physiological functions of cartilage and subchondral bone simultaneously [19].

In this study, we fabricated bilayered PLGA scaffolds (Figs. 1 and 2) to restore the osteochondral defects of rabbits. The fabrication of bilayered scaffolds constituted two basic steps: first, to obtain porous foams for either cartilage layer or bone layer by porogen leaching and cold compression; then, to stick the two layers together by a versatile organic solvent. We would like to call this approach to fabricate integrated bilayered scaffolds as a porogen-leaching and solvent sticking one.

Osteochondral defects with 4 mm in diameter and 5 mm in depth were created in rabbit knee joints, and then the bilayered scaffolds were implanted into the defects. It has been known that the critical size for cartilage defects in a rabbit model is 3 mm [44]. This was confirmed by our study that the defects with 4 mm diameter were not self-repaired, as shown in Figs. 5d, 6b and 7b. A gap was obvious in the defects, and the little regenerative tissues were filled mostly by fibrocartilage (Fig. 7b and d). In contrast, our bilayered scaffold implantations stimulated regeneration of osteochondral defects, as seen from the macroscopic appearances (Fig. 6) and histological scores (Fig. 9) of the regenerative tissues. Incorporation of BMSCs into the scaffolds achieved better restoration of osteochondral defects. The implanted BMSCs seemed beneficial especially for the cartilage regeneration, and the DiI-labelled cells were still observed in the neo-tissue in our fluorescence observation (Fig. 4).

The regenerative cartilage integrated well with the adjacent host tissues (Fig. 5a), and even the border could not be easily identified (Fig. 6a). According to the images after H&E staining (Fig. 7a and c) and immunohistochemical staining (Fig. 8c and d), the thickness of the regenerative cartilage was appropriate, and the cells exhibited the lacuna morphology, reminiscent of the hyaline cartilage. The tidemark between cartilage and subchondral bone was also clearly visualized after the toluidine blue staining, as shown in Fig. 8b. The upper layer was collagen II rich as an indicator of cartilage, and the lower layer was rich in collagen I consistent with the subchondral bone (Fig. 8c and d).

### The porosity effect on osteochondral repair using bilayered scaffolds is different from that on merely cartilage or bone repair using single-layer scaffolds

Although porosity is a very basic parameter of tissue engineering scaffolds, the porosity effect of bilayered scaffolds on osteochondral repair has never been examined according to our survey.

The porosity effects for merely cartilage or bone repairs have been reported; yet the discussion is of diversity. For cartilage regeneration, Sherwood *et al.* [45, 46] illustrated that the scaffold should have a porosity as high as 90% to facilitate cell attachment, proliferation and matrix deposition; Xie *et al.* [47] indicated that the scaffold of a low porosity (71%) appeared to be suitable for cartilage repair if the mechanical properties were closer to those of native cartilage tissues. For bone regeneration, it is believed that a high porosity always benefit for bone ingrowth and formation [48, 49], and meanwhile some reports found no significant effect of porosity on the amount of apposite bone [50]. In a wonderful review focused on porosity effects, Karageorgiou and Kaplan [21] summarized ‘*in vivo*, higher porosity and pore size result in greater bone ingrowth, a conclusion that is supported by the absence of reports that show enhanced osteogenic outcomes for scaffolds with low void volumes’. Ikeda *et al.* [28] fabricated single-layer synthetic polymer scaffolds to examine the effect of porosity on repair of osteochondral defects and found that the scaffold with higher porosity allowed better migration of bone marrow cells and better repair of bone and cartilage.

The claims summarized above are for single-layer scaffolds. For the bilayered scaffold as focused in this study, one should take the porosity pair (the cartilage layer versus the subchondral bone) into consideration. Although porosities varied, pore sizes were fixed among 200–300 μm in this study, which is reasonable for the restoration of cartilage and bone [21]. Three porosity combinations were designed, as listed in Table 1. Among the three pairs, scaffold A (92% in upper layer and 77% in down layer) exhibited the best repair efficacy (Figs. 5a, 6a, 9 and 10). As the osteogenesis is concerned, our studies offer a report that shows enhanced osteogenic outcomes for scaffolds with low porosity, different from the previous summary by Karageorgiou and Kaplan [21]. The different conclusions do not contradict with each other, because herein bilayered scaffolds were examined.

Then how to understand that the cartilage layer favours the high-porosity scaffold and the subchondral layer prefers the low-porosity scaffold? The key might be the biomechanic matching. Cartilage is a highly hydrated composite with a relatively low compressive stiffness, whose instantaneous compressive Young’s modulus is 1.36–39.2 MPa [51–53]. The subchondral bone is a thin layer of bone contacting the articular cartilage; and the cancellous bone is of compressive modulus among 1.4–9800 MPa [54, 55]. Scaffold A has a porosity of 92% in the upper layer and of 77% in the lower layer. The upper layer for cartilage has good interconnectivity (Fig. 2c and f) but low compressive modulus (Fig. 3). In the compression test, the deformation happened first from the highly porous and major part, and then the dense part. Two slopes were seen in the stress–strain curve of scaffold A. *E*1 is for the upper layer and the value is 3.8 ± 1.1 MPa, which is well compatible for the cartilage. For the lower layer, *E*2 (29.1 ± 5.0 MPa) is higher than *E*1, which might favour the bone ingrowth. The mechanical properties in the two layers in scaffold C are reversed to those of scaffold A, and thus the regenerative efficacy was relatively the worst. Scaffold B with the same porosity in the two layers resulted in a moderate regenerative outcome. So, it was the compatible mechanical property of scaffold A that led to its very good restoration efficacy.

## Conclusions

Bilayered PLGA scaffolds were made via a porogen-leaching and solvent-sticking approach. Three combination groups of porosities were examined in the rabbit model using BMSCs as seeding cells. All the bilayered scaffolds with porosities among 77–92% were found to repair the osteochondral defects (4 mm in diameter and 5 mm in depth) in the femoral condyle quite well after 12 weeks. According to cross-section view, H&E staining, toluidine blue staining, immunohistochemical staining, histological scores and relative gene expression levels of reparative tissues by real-time PCR, scaffold A with 92% porosity in the cartilage layer and 77% porosity in the subchondral bone layer exhibited the best efficacy. The porosity and thus the modulus of scaffolds should match the biomechanics of repaired tissues, which is especially important for more-than-one tissues connecting together. This article examines the porosity effect in a fixed pore size. We have another publication about the pore size effect at a fixed porosity [56]. The optimal porosity might depend on pore sizes, matrix material types, animal types and other factors. So, the concrete values might vary with cases. Yet the porosity effect is definitely worthy of taking into consideration in design of tissue engineering scaffolds.

## Funding

This work was supported by Chinese Ministry of Science and Technology (973 Programs No. 2009CB930000 and No. 2011CB606203), National Science Foundation of China (Grant No. 21034002, 31170925, and 51273046), Science and Technology Developing Foundation of Shanghai (Grant No. 13XD1401000) and Shanghai International Science and Technology Partnership Program (No. 11540702700).

*Conflict of interest statement*. None declared.
